# Clinical practice guidelines on the use of deep brain stimulation for the treatment of obsessive–compulsive disorder: systematic review

**DOI:** 10.1192/bjo.2023.539

**Published:** 2023-08-08

**Authors:** Adele Mazzoleni, Shreya Bhatia, Maria A. Bantounou, Niraj S. Kumar, Monika Dzalto, Roy L. Soiza

**Affiliations:** Barts and the London School of Medicine and Dentistry, UK; Queen's University Belfast, Northern Ireland; School of Medicine, Medical Sciences and Nutrition, University of Aberdeen, Aberdeen, UK; and National Medical Research Association, London, UK; National Medical Research Association, London, UK; Brighton and Sussex Medical School, UK; School of Medicine, Medical Sciences and Nutrition, University of Aberdeen, Aberdeen, UK

**Keywords:** AGREE II, clinical practice guidelines, deep brain stimulation, obsessive–compulsive disorder, systematic review

## Abstract

**Background:**

Deep brain stimulation (DBS) has been proposed to improve symptoms of obsessive–compulsive disorder (OCD) but is not yet an established therapy.

**Aims:**

To identify relevant guidelines and assess their recommendations for the use of DBS in OCD.

**Method:**

Medline, Embase, American Psychiatric Association PsycInfo and Scopus were searched, as were websites of relevant societies and guideline development organisations. The review was based on the PRISMA recommendations, and the search strategy was verified by a medical librarian. The protocol was developed and registered with PROSPERO (CRD42022353715). The guidelines were assessed for quality using the AGREE II instrument.

**Results:**

Nine guidelines were identified. Three guidelines scored >80% on AGREE II. ‘Scope and Purpose’ and ‘Editorial Independence’ were the highest scoring domains, but ‘Applicability’ scores were low. Eight guidelines recommended that DBS is used after all other treatment options have failed to alleviate OCD symptoms. One guideline did not recommend DBS beyond a research setting. Only one guideline performed a cost-effectiveness analysis; the other eight did not provide details on safe or effective DBS protocols.

**Conclusion:**

Despite a very limited evidence base, eight of the nine identified guidelines supported the use of DBS for OCD as a last line of therapy; however, multiple aspects of DBS provision were not addressed.

Obsessive–compulsive disorder (OCD) is a chronic mental illness characterised by obsessions (intrusive thoughts) and compulsions (ritualistic behaviours). These two characteristics are present in healthy people, but in OCD they are prevalent enough to affect social, occupational and personal areas of the individual's life.^1^ As OCD symptoms exhibit heterogeneity, the applicability of a general pathophysiological model has been questioned. Nevertheless, most neuroimaging and neuropsychological studies have found that dysfunction of a cortico-striato-thalamo-cortical circuit plays a crucial part in symptom development.^2^ Furthermore, the prevalence of OCD varies among regions and is estimated to affect between 0.8 to 1.3% of the population worldwide.^3^ The mean age of onset is approximately 20 years, with two known peaks at the ages of 9 to 11 and 20 to 23 years, and another third peak having been suggested to start after the age of 65 years.^4,5^ This latter cohort of patients is commonly overlooked, with only 10% seeking consultations for their symptoms.^6^

Three main modalities can be used for the treatment of OCD: pharmacotherapy, psychotherapy and neuromodulation. Pharmacotherapy, namely selective serotonin reuptake inhibitors (SSRIs), and psychotherapy are offered as first-line treatments;^1^ however, improvement is seen in approximately 70 and 64% of patients, respectively.^7^ There are not enough data to show remission rates for pharmacotherapy–psychotherapy combination therapy, which is nonetheless oftentimes used as it has been found to be more effective than pharmacotherapy alone, especially in severe cases of OCD.^8^ If first-line treatments, including the combination of psychotherapy and SSRI pharmacotherapy, fail to generate a response, the condition is termed treatment-refractory OCD. In such cases, antipsychotic therapy may be recommended in combination with an SSRI, producing a response rate of 29.8%. However, this can have significant adverse effects and is therefore discontinued if not effective within 6–10 weeks.^9,10^

If antipsychotic therapy is deemed to be ineffective, neuromodulation therapy may be offered, namely deep brain stimulation (DBS). DBS is a type of invasive neurosurgical treatment that uses electrodes implanted in target areas of the brain and an implantable pulse generator device, which delivers stimulation to these areas. Traditionally, DBS has been used for the treatment of neurological movement disorders, but more recently it has also been implemented for the treatment of psychiatric disorders, including OCD.^11^ The structures in the brain that have been targeted for OCD are the anterior limb of the internal capsule, subthalamic nucleus and nucleus accumbens in the ventral striatum, the latter being the preferred target. Studies have demonstrated that DBS as a treatment for OCD can produce a wide variety of response rates, ranging from 10 to 61%. This is largely owing to differences in neuroanatomical placement of electrodes and types of electrode and stimulation.^12^

According to their consensus guidelines published in 2021, the World Society for Stereotactic and Functional Neurosurgery considers DBS to be an emerging but not yet established treatment, owing to current evidence not meeting their standards.^13^ Nevertheless, some countries have accepted the available evidence and have approved DBS for use in treatment of refractory OCD. Notably, the US Food and Drug Administration approved DBS for severe OCD in 2009. The same year, DBS also received a Conformité Européenne (CE) mark, which indicates that the procedure is safe for use, although it does not provide any assurances about its efficacy. Subsequently, the procedure was approved and implemented in a number of EU countries.^14^

Overall, there are multiple treatment options for OCD, each appropriate for different degrees of symptom severity. The designated institutions of different countries have developed guidelines to provide evidence-based recommendations to relevant healthcare providers on safe, effective and up-to-date treatment options for OCD. These guidelines often vary from country to country and sometimes even among institutions within countries, with inconsistencies and contradictions observed among recommendations.

Therefore, the primary aim of this review was to identify OCD guidelines on a global scale and assess their recommendations for the use of DBS in OCD. The quality of each guideline was graded to help determine the place of DBS in OCD treatment. The secondary aim was to determine whether treatment recommendations were tailored to individual patient characteristics, such as age, gender and other comorbidities.

## Methods

### Search strategy

A systematic search of published guidelines and recommendations on the use of DBS for the treatment of OCD was undertaken using relevant synonyms for the terms ‘guideline’, ‘DBS’ and ‘OCD’. The protocol was developed and registered with PROSPERO under registration number CRD42022353715. An initial search was performed on the MEDLINE, Embase, American Psychiatric Association (APA) PsycInfo and Scopus databases from inception to 29 July 2022. The initial strategy was modified on 22 October after consultation with a medical librarian; a detailed overview of the final and verified search strategy can be found in Appendix 1. In addition, websites of relevant societies and guideline development organisations, as presented in Appendix 2, were searched to identify all relevant records. This systematic review was designed, conducted and reported according to PRISMA (Preferred Reporting Items for Systematic Reviews and Meta-Analyses) recommendations.^13^

### Inclusion and exclusion criteria

OCD treatment guidelines and evidence-based recommendations that included information on DBS were included in this review. DBS guidelines on psychiatric conditions that provided recommendations for OCD were also assessed for eligibility and inclusion in this study. The search was restricted to guidelines developed from evidence-based searches or expert opinions and/or consensus that were written in English. In instances where a single organisation published multiple guidelines on the same topic, only the most recent one was included. Records that did not mention DBS as part of their treatment recommendations, were not written by a professional body, were in a draft form or only had an abstract available were excluded from this review.

### Study selection

Studies were assessed and selected by two independent reviewers (S.B. and A.M.) based on pre-specified inclusion and exclusion criteria. This was done in two stages: (a) by title and abstract screening and (b) by full-text screening. Studies that both reviewers agreed were ineligible were excluded at both stages. Discrepancies were managed by a third reviewer (M.B.) acting as an adjudicator. Websites of relevant societies and guideline development organisations were also identified and searched by two reviewers independently.

### Data extraction

Data were extracted and collected by two reviewers (S.B. and A.M.) independently using a predefined Excel data extraction form. Characteristics of the practice guidelines, details of the relevant recommendations and data that were needed for the guideline's quality assessment according to the AGREE II instrument^14^ were extracted.

The AGREE II instrument was used by the two reviewers independently to grade the quality of each guideline. This is a 23-item standardised tool that assesses the quality of a guideline based on six key domains: (a) scope and purpose, (2) stakeholder involvement, (3) rigour of development including evidence base, (4) clarity of presentation, (5) applicability and (6) editorial independence. Each domain was scored on a scale from 1 (lowest quality) to 7 (highest quality) by both reviewers, with combined scores for each domain and guideline calculated using the ‘My AGREE PLUS’ platform. The following formula was used to determine the scaled domain percentage score: (obtained score − minimum possible score) / (maximum possible score − minimum possible score), where ‘obtained score’ is the sum of the reviewers’ scores for each assessed item and as such considers discrepancies in scoring between the reviewers. Two domains of the AGREE II instrument, ‘stakeholder involvement’ and ‘editorial independence’, were used to indicate the risk of bias of the included guideline, with the scores for these domains reflected in the overall score for the guideline. As per AGREE II, stakeholder involvement refers to the extent to which guidelines have been produced by the appropriate stakeholder and include the point of view of the intended users. Editorial independence ensures that the guideline has been developed independently from the funding body, and that competing interests have been appropriately addressed.

For guidelines focusing on DBS alone, item 16 (management options) of the AGREE II instrument was rated as either 1 or 2 in accordance with AGREE II guidance.^14^ If the AGREE II scores varied by more than three points between the two reviewers, a third reviewer adjudicated the outlier scores.

### Data synthesis

The findings of this systematic review of guidelines were synthesised narratively. All included guidelines were critically appraised and graded using the AGREE II scoring system. Results of this analysis were cross-tabulated, allowing for comparisons of the guidelines.

## Results

### Guidelines selection

**Fig. 1:**
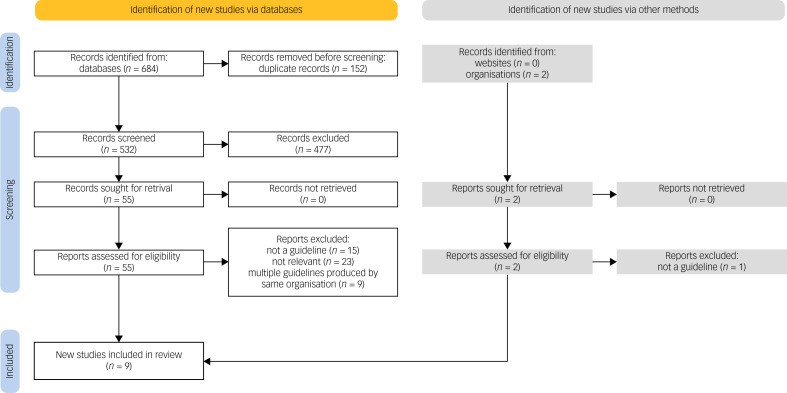
PRISMA flow diagram showing the process followed for selection of eligible papers, including the numbers of included papers identified via database search and hand-search of grey literature. For the grey literature search, 20 of the 22 reports were not retrieved as the respective websites and/or organisations did not produce relevant OCD treatment guidelines.

The initial literature database search and hand-search yielded 684 records. Following deduplication, 532 papers were retrieved for title and abstract screening. At this point, 477 papers were excluded according to the study's inclusion and exclusion criteria. The full texts of the remaining 55 papers were assessed. Among these, eight were eligible for inclusion: five from OVID and three from Scopus. In addition, 11 websites and the webpages of 11 organisations were searched for identification of relevant guidelines to be included in this review. Of the 22 websites and organisations, only two had available guidelines; these were assessed for inclusion in the study according to the eligibility criteria. The complete list of additional websites and organisations that were searched can be found in Appendix 2. A summary of the record selection process and reasons for record exclusion can be found in Fig. 1.

### Key recommendations on DBS

Table 1 presents the main characteristics of the included guidelines. Specifically, three were developed and published in the USA,^13,15,16^ two in the UK,^17,18^ one in Canada,^19^ one in Brazil,^20^ one in India^21^ and one by the World Federation of Societies of Biological Psychiatry (WFSBP).^22^

**Table 1:** Characteristics of guidelines that provide a recommendation on the use of DBS for the treatment of OCD, including the authors, organisation and countries involved in guideline development, the target users, the included evidence and the guideline scope

Guideline authors and year of publication	Guideline name	Organisation and country of development	Target users	Included evidence	Guideline scope
Beaulieu et al., 2019	The Psychopharmacology Algorithm Project at the Harvard South Shore Program: an algorithm for adults with OCD.	Harvard South Shore, USA	Clinicians (psychiatrists)	Meta-analysis of 31 DBS trials	Pharmacological and non-pharmacological treatments for OCD in adults
Reddy et al., 2017	Clinical practice guidelines for OCD	Indian National Institute of Mental Health & Neurosciences, India	Clinicians (psychiatrists)	Sham controlled studies; one meta-analysis	Pharmacological and non-pharmacological treatments for OCD in adults
Staudt et al., 2020	DBS for OCD	American Association of Neurological Surgeons/Congress of Neurological Surgeons, USA	Clinicians and surgeons	One class 1 study and one class 2 study	Provides recommendations on the use of DBS for OCD in adults
Bernardo et al., 2018	DBS for depression and OCD.	Brazilian Neurosurgery Society, Brazil	Clinicians (psychiatrists)	Case reviews, randomised, double-blinded, phase II clinical trials (21 total)	Reviews the use of DBS in depression and OCD
Katzman et al., 2014	Canadian Clinical Practice Guidelines for the management of anxiety, PTSD and OCD	Anxiety Disorders Association of Canada, Canada	Primary care physicians, psychiatrists, psychologists, social workers, occupational therapists and nurses	One pilot study using staggered-onset design; one short-term blinded, on–off design and long-term open follow-up; one case review	Reviews the clinical features and management of anxiety and related disorders
Koran et al., 2007	Practice guideline for the treatment of patients with OCD	American Psychiatric Association, USA	Clinicians (psychiatrists)	Two double-blinded trials, several case reports	Reviews the clinical features and management of OCD
NICE, 2021	DBS for chronic, severe, treatment-resistant OCD in adults	National Institute for Health and Care Excellence, UK	Experts in psychiatry, neuropsychiatry, clinical psychology, neurology, neurosurgery and DBS	Two randomised controlled trials, two meta-analyses, two systematic reviews, one non-randomised comparative study, four case series and one case report	Reviews the data on DBS for OCD
Baldwin et al., 2014	Evidence-based pharmacological treatment of anxiety disorders, PTSD and OCD: a revision of the 2005 guidelines from the British Association for Psychopharmacology	British Association of Psychopharmacology, UK	Primary, secondary and tertiary medical care, patients, carers, medicines management and formulary committees.	Three case reports	Provides recommendations on the pharmacological (and non-pharmacological, partly) treatment of anxiety disorder, PTSD and OCD
Bandelow et al., 2022	World Federation of Societies of Biological Psychiatry guidelines for treatment of anxiety, OCD and PTSD – version 3. Part II: OCD and PTSD	World Federation of Societies of Biological Psychiatry	Clinicians (psychiatrists)	Thirteen open retrospective and prospective studies	Review of the data on pharmacological and non-pharmacological treatments for PTSD and OCD

DBS, deep brain stimulation; OCD, obsessive–compulsive disorder; PTSD, post-traumatic stress disorder.

Overall, all guidelines recommended the use of DBS after all other treatment options have failed to alleviate OCD symptoms,^13,15,16,18–22^ with the exception of the UK's National Institute for Health and Care Excellence (NICE) guideline; this was the only one to recommend DBS for use in research settings rather than clinical practice.^17^ DBS settings were not specified in any of the included guidelines, with a recommendation on target regions made only by the Congress of Neurological Surgeons (CNS) guideline. They proposed and included the subthalamic nucleus or nucleus accumbens as the electrode placement region.^13^ Key recommendations on the use of DBS for OCD, including details on DBS settings and specifics of the place of DBS in the treatment of OCD as recommended by the guidelines, can be found in Table 2.

**Table 2:** Key recommendations on the use of DBS for OCD from selected guidelines, including details on DBS settings and specifics of the place of DBS in the treatment of OCD

Guideline	Key recommendation on DBS	Details on DBS settings	Place of DBS in treatment of OCD	AGREE II overall score (%)
HSSP, 2019	DBS remains experimental. Only recommended if inadequate response to pharmacological treatment and non-invasive therapy.	Optimal brain region is still being established.	After inadequate response to pharmacological treatment (SSRI, second generation anti-psychotics, novel agents) and non-invasive therapy (transcranial magnetic stimulation).	58
NIMHANS, 2017	DBS recommended in carefully selected patients with treatment-refractory OCD after discussion.	Optimal brain region is still being established.	After inadequate response to pharmacological treatment (SSRI, clomipramine), cognitive–behavioural therapy, and repetitive transcranial magnetic stimulation and/or transcranial direct current stimulation.	58
CNS, 2020	Bilateral subthalamic nucleus and/or bilateral nucleus accumbens DBS can be recommended for medically refractory OCD. Insufficient evidence to make a recommendation for unilateral treatment.	To be performed bilaterally in the subthalamic nucleus and nucleus accumbens.	After inadequate response to pharmacological treatment.	92
BNS, 2018	Patients with depression or OCD refractory to appropriate forms of treatment are considered for DBS.	No recommendations.	After inadequate response to pharmacological treatment (SSRI, clomipramine) and cognitive–behavioural therapy.	67
ADAC, 2014	DBS is the recommended third-line treatment for refractory OCD.	Optimal brain region is still being established.	After inadequate response to pharmacological treatment (SSRIs, clomipramine, anti-depressants, adjunctive therapy) and cognitive–behavioural therapy.	58
APA, 2007	No definitive conclusions on DBS recommendations owing to limited number of studies, but it can be considered as third-line therapy.	No recommendations.	After inadequate response to pharmacological treatment (SSRI, augmentation strategies) and cognitive–behavioural therapy.	83
NICE, 2021	Evidence on DBS for OCD is inadequate. This procedure should only be used for research.	N/A	Only for research purposes.	83
BAP, 2014	Some patients with treatment-refractory OCD may benefit from DBS.	No recommendations.	After inadequate response to pharmacological treatment (SSRI, antipsychotics, Clomipramine, augmentation strategies) and cognitive behavioural therapy.	50
WFSBP, 2022	DBS should only be used in patients with chronic, severe and treatment-resistant OCD.	No recommendations.	After inadequate response to pharmacological treatment (SSRI, clomipramine, augmentation strategies) and cognitive behavioural therapy.	58

DBS, deep brain stimulation; OCD, obsessive–compulsive disorder; N/A, not applicable; SSRI, selective serotonin reuptake inhibitor.

### Overview of AGREE II scores

The overall AGREE II scores varied among guidelines. Only the CNS, APA and NICE guidelines had average score higher than 80%,^13,16,17^ indicating that they are of high quality.^23–25^ The Brazilian Neurosurgery Society (BNS) guidelines had a score of 67%, which is considered indicate sufficient quality.^23–25^ The remaining five guidelines, i.e. those of the Harvard South Shore Program (HSSP), National Institute of Mental Health and Neurosciences (NIMHANS), Anxiety Disorder Association of Canada (ADAC), British Association of Psychopharmacology (BAP) and WFSBP, had scores that fell between 50 and 58%.^15,18,19,21,22^ These five guidelines with the lowest scores were not focused on DBS but on pharmacological or alternative options, and as such the reviewers did not consider that sufficient evidence was provided for the DBS recommendations made.

**Fig. 2:**
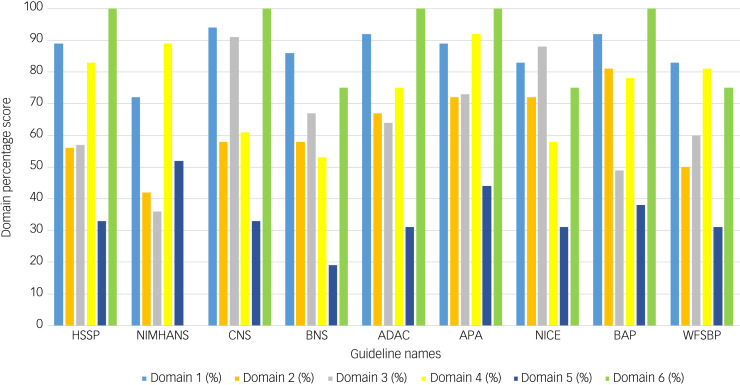
Bar graph of AGREE II domain scores for the nine included guidelines. Domain 1 (light blue) indicates scope and purpose, domain 2 (orange) indicates stakeholder involvement, domain 3 (grey) indicates rigour of development, domain 4 (yellow) indicates clarity of presentation, domain 5 (dark blue) indicates applicability and domain 6 (green) indicates editorial independence. Calculated percentage scores, as specified in the methodology and according to the AGREE II guidance, per domain for each guideline are presented on the y-axis of the bar chart. Guideline names are shown on the x-axis.

The guideline with the highest overall AGREE II score of 92% was the CNS guideline, whereas the lowest-scoring guideline with an overall AGREE II score of 50% was the BAP guideline (Table 2). Domains 1 (scope and purpose) and 6 (editorial independence) scored consistently high among the nine guidelines, with average scores of 87% and 81%, respectively (Fig. 2). Overall, all guidelines provided clear summaries of their aims and purposes, while also demonstrating editorial independence. Domains 5 (applicability) and 2 (stakeholder involvement) scored the lowest among the six domains, with average scores of 35 and 62% across all nine guidelines, respectively (Fig. 2). Table 2 provides the overall AGREE II scores for each guideline as percentages, whereas Fig. 2 illustrates the breakdown of the AGREE II percentage scores for each individual domain. A detailed AGREE II quality appraisal for each domain of every included guideline can be found in Appendix 3, and an overview of all percentage scores for the guidelines can be found in Appendix 4.

### Summary of similarities and differences among guidelines

All guidelines included adult patients diagnosed with OCD according to DSM-5 as the target population.^13,15–22^ For the most part, cost considerations were not thoroughly considered as part of the analysis; the NICE guidelines stated that a health economic analysis was performed as part of the guideline development process but did not mention specific costs of DBS.^26^ The NIMHANS only mentioned that DBS has high costs, especially during maintenance, but included no data or further details.^21^ Apart from these, no guidelines mentioned a cost analysis. Furthermore, none of the guidelines sought patient preferences when formulating their recommendations. None of the guidelines had recommendations for specific age groups, ethnicities or OCD patients with comorbidities.

The APA, NICE, BAP and WFSBP guidelines mentioned their general updating process on their website; however, three (APA, BAP and WFSBP) lacked details on specific timeframes scheduled for the update.^16,18,22^ The types of included evidence also varied across guidelines (Table 2). The CNS guideline included evidence from clinical randomised controlled trials (RCTs; class 1) and a case–control study (class 2).^13^ The BNS, APA, NICE and WFSBP guidelines also all included RCTs as part of the reported evidence. The BAP and ADAC guidelines used level 4 evidence, which includes evidence from case–control or cohort studies, to construct their recommendations.

## Discussion

This study is the first systematic review of available guidelines on the use of DBS for treatment of OCD. We found that eight of nine guidelines recommended using DBS in the treatment of OCD as a last line of therapy, whereas one did not recommend its use in clinical practice. Overall, none of the guidelines provided extensive recommendations for DBS settings, with most referring to settings and target areas explored in the literature. Nevertheless, one guideline, the CNS, recommended the subthalamic nucleus and nucleus accumbens as target regions for stimulation. Furthermore, this guideline had the highest overall AGREE II score of 92%, indicating that it is of high methodological quality. The domains ‘scope and purpose’ and ‘editorial independence’ were the highest scoring across all guidelines, whereas ‘applicability’ and ‘stakeholder involvement’ were the lowest scoring. Applicability in the AGREE II scoring system refers to possible barriers or facilitators to the use of the guideline and the potential cost considerations when implementing the measure in question, in this case DBS. It also includes monitoring criteria and advice on to how to put the guideline into practice.^25,27^ The low applicability scores across the guidelines indicate that, overall, costs were not taken into account, but also that practical ways to implement DBS for OCD were not considered. Among the examined guidelines, only NICE involved a health economist in the cost analysis of the recommendation. This may be among the reasons that NICE was the only guideline to recommend against using DBS. It was also noted that although NICE and BAP are both UK-based guidelines, they had different recommendations about DBS.

Failure to consider the cost of DBS implementation can be problematic. With the total operative and device costs per patient treated with DBS estimated to be between $27,497 and $35,531,^28^ the financial implications of recommending DBS for OCD need to be transparently presented and thoroughly considered by healthcare bodies and other relevant stakeholders prior to implementation and provision. As such, performing a quality-adjusted life-year analysis or similar cost–benefit analysis is a key area for future research endeavours. Other pivotal limitations identified across all nine guidelines were lack of information on the contribution of each member to the development of the guideline and lack of detailed time-specific guideline-updating procedures.

Notably, across all guidelines, there was a lack of patient stratification and consideration of individual patient characteristics such as age and ethnicity. The only exception to this was the HSSP guideline, which mentioned that patients with specific obsessions towards sexual or religious content and those of older age responded better to DBS. In addition, patients with OCD commonly present with co-existing conditions such as depression and anxiety. Thus, further studies that include OCD patients with comorbid disease should be conducted to determine the effects of DBS on OCD-associated comorbidities, with guidelines updated to reflect such evidence.^29^ Therefore, more precise patient stratification based on factors such as comorbidities, age, ethnicity and OCD symptoms may lead to improvements in treatment outcomes. This has been demonstrated by the use of DBS for the treatment of Parkinson's disease, where improved screening and selection of candidates to undergo DBS has resulted in higher levels of safety and efficacy.^30,31^

DBS is a technique that is becoming increasingly popular. It is being explored and offered as a treatment for a range of conditions, including psychiatric conditions such as depression and OCD, and movement disorders.^32^ For OCD specifically, this is a new intervention with a limited amount of published literature. Fewer than 500 patients had reportedly received DBS for the management of OCD across the world as of 2020, as reported by Denys and colleagues, with many of the published studies having a small sample size (five patients on average).^33,34^ Prior to DBS, ablative surgery was considered as a treatment option for a number of neurological and psychiatric disorders. Compared with that procedure, DBS has the benefits of being reversible and less destructive.^32^ Nonetheless, DBS is still a neurosurgical intervention and comes with several risks including brain haemorrhage, infection, seizures,^21,33^ irritability, increased anxiety levels and insomnia.^29^ Changes to cognition and impaired concentration have also been reported.^30^ Despite this, just three guidelines have considered the DBS side-effect profile: BNS, APA and WFSBP. The BNS guidelines stated that adverse effects of stimulation have included transient sadness, anxiety, vertigo, euphoria, and motor and olfactory symptoms. However, these symptoms were reversible once stimulation was stopped. APA outlined brain haemorrhage, infection and seizures as risks of DBS. WFSBP highlighted the importance of considering the adverse effects. As such, the application of DBS to neuropsychiatric disorders has been debated; this is possibly among the reasons that large-scale clinical trials have been somewhat limited.^35^

In addition, guidelines did not provide recommendations on the perioperative care required for patients. Indeed, a patient should receive a full pre-operative assessment prior to DBS, including adequate education regarding to the procedure.^32^ Furthermore, post-operative management should also be established. Currently, there is a lack of standardised setting programming for DBS for the treatment of OCD, and refined adjustable DBS parameters have not yet been agreed, generating variability in practice. By contrast, the settings for DBS for Parkinson's disease have been tailored and established to safe and effective margins.^32,36^

With clinical guidelines increasingly influential in the past few decades,^37,38^ a number of concerns have been reported with the guideline writing process, which we also found to apply to DBS for OCD. There was great variability in guideline quality, reflected in the overall AGREE II scores that spanned from 50 to 92%. There was also considerable variability in the type and quality of evidence used to develop the recommendations. In line with this, it appears that more than half of guidelines developed did not use systematic methods to build their recommendations, a problem which undermines their credibility and may also lead to misleading information for clinicians and surgeons.^38^ A lack of precise and transparent guideline-updating processes is a further factor that affects the integrity of guidelines. Notably, 72% of institutions have self-reported that their guideline-updating process could be more rigorous.^37^ Living guidelines have been suggested in response to this; however, these are too labour-intensive and resource-consuming.^37^ This review included guidelines published from 2007 to 2022. Older guidelines that did not include more recent evidence were included in this review as they are still used in practice. This highlights the need for regular guideline-updating parameters.

Recommendations for the optimal brain region to target for stimulation are also yet to be established, as are those for specific voltages and frequency of stimulation.^39,40^ There is a lack of RCTs in this field, and further research is called for to improve the quality of evidence available and consequently the quality of recommendations.^32^ Furthermore, owing to the financial implications of providing DBS treatment for OCD, a rigorous cost analysis is needed. Finally, DBS should be offered at an individual patient level, based on patient characteristics. Notably, females^3^ and the elderly population^4^ are at higher risk of developing OCD, and psychiatric comorbidities and polypharmacy are associated with worse OCD outcomes and poorer quality of life.^41^ The response to DBS of such patient cohorts may differ from that of other patients with OCD. As the evidence used for guideline development is derived from RCTs, a systematic review of RCTs stratifying the safety and efficacy of DBS in clinically relevant groups of patients would be of value. This would allow guidelines to provide more precise recommendations. No such gaps in the literature were highlighted by any of the included guidelines, possibly in efforts to provide recommendations that can be applicable to all patients^42^ and adjusted on a case-by-case basis according to clinical judgement. Thus, future work on stratifying eligible patients needs to be undertaken to determine whether DBS for OCD is more efficacious in a sub-population of patients.

This study had some limitations. First, the AGREE II scoring system does not provide predefined cut-offs for low- or high-quality guidelines. These were set by the review team and were in accordance with previously published literature. Furthermore, the AGREE II scoring system does not rely primarily on the quality of the evidence the recommendations are based on, but on the overall design and development of the guideline in question. Therefore, a guideline could have a low overall AGREE II score despite its recommendation being based on high-quality evidence. Although the authors of this review hand-searched relevant websites of organisations and bodies, as well as systematically searching scientific literature databases, it is possible that some relevant guidelines that would have been eligible for inclusion in this study were missed. However, hand-searching of bibliographies revealed only one additional guideline. In addition, most of the guidelines were based in high-income countries with a high prevalence of Caucasian people, with only two having been developed in middle-income countries (i.e. Brazil and India). This demonstrates a lack of perspective on the uses of DBS for OCD in low-income countries. It is noteworthy, however, that DBS is currently a technique that is not largely available in low- and middle-income countries. Furthermore, the clinical practice guidelines did not provide treatment recommendations according to individual patient characteristics, nor information on the clinical and demographic characteristics of patients to whom DBS should be offered.

Nevertheless, this study is the first systematic review of available guidelines on the use of DBS for OCD and has shown that numerous guidelines have already been published despite the paucity of evidence. A hand search was performed in addition to searching literature databases. Thus, guidelines that could have potentially been missed in the database search were captured and included in this review. In addition, a quality appraisal was conducted using the AGREE II instrument, a standardised tool specifically constructed to report guideline quality. Objectivity and validity were thus added to the assessment undertaken, as well as replicability.

## Conclusions

Applicability and a lack of health economic appraisal were identified as key areas of concern across guidelines. Furthermore, transparency in update procedures, cost analyses and recommendations for specific DBS settings were areas identified as either underreported or not reported at all. Further work is still needed, particularly focusing on providing DBS recommendations that consider individual patient characteristics and comorbidities. Therefore, although eight of the nine identified guidelines supported the use of DBS for OCD as a last line of therapy, owing to the lack of information on many aspects of DBS, we believe that additional high-quality evidence is required prior to DBS being widely implemented.

## Data Availability

Data availability is not applicable to this article as no new data were created or analysed in this study.

## Appendices

### Appendix 1: Search strategy

**SCOPUS:**

TITLE (guid* OR (recommend*) OR (clinical AND practice AND guid*)) AND (ocd OR (obsessive AND compulsive AND disorder*)) AND (dbs OR (deep AND brain AND stimulation*))

**OVID (MEDLINE, APA PsycInfo AND Embase):**

**Obsessive-Compulsive Disorder**

exp Obsessive-Compulsive Disorder or exp Obsessive Compulsive Disorder OR (OCD or obsessive-compulsive disorder$ or obsessive compulsive disorder$).

AND **Guideline**

exp Practice Guideline or exp Guideline or (recommend$ or guid$).mp.

AND **Deep Brain Stimulation**

Deep Brain Stimulation OR (DBS or deep brain stimulation$).mp.

## Appendix 2: Websites of organisations responsible for guidelines

- Guideline International Network https://g-i-n.net/
- Association of the Scientific Medical Societies in Germany https://www.awmf.org/en/clinical-practice-guidelines.html
- International Database of GRADE Guidelines https://sites.bvsalud.org/bigg/en/biblio/
- GRADEpro https://guidelines.gradepro.org/search
- Dynamed https://www.dynamed.com/
- Ebmafrica http://www.ebmafrica.net/
- ECRI Guideline Trust https://guidelines.ecri.org/
- Dec.gov.ua https://dec.gov.ua/en/
- Trip Database https://www.tripdatabase.com/
- Agency for Healthcare Research and Quality https://www.ahrq.gov/prevention/guidelines/index.html
- SIGN https://www.sign.ac.uk/
- World Health Organization https://www.who.int/
- Royal College of Psychiatrists https://www.rcpsych.ac.uk/
- European Psychiatric Association https://www.europsy.net/
- NICE https://www.nice.org.uk/guidance/ipg693/resources/deep-brain-stimulation-for-chronic-severe-treatmentresistant-obsessivecompulsive-disorder-in-adults-pdf-1899874404226501#:~:text=Treatment%20options%20include%20psychological%20interventions,usually%20selective%20serotonin%20reuptake%20inhibitors).&text=Deep%20brain%20stimulation%20for%20OCD,stereotactic%20frame%20may%20be%20used.
- National Institute of Mental Health https://www.nimh.nih.gov/
- Royal Australian and New Zealand College of Psychiatrists https://www.ranzcp.org/files/resources/college_statements/clinical_memoranda/cm-deep-brain-stimulation.aspx
- The Royal Netherlands Academy of Arts and Sciences https://pure.knaw.nl/portal
- Asian Federation of Psychiatric Associations http://www.afpa.asia/en/index.html
- African Association of Psychiatrists and Allied Professions https://uia.org/s/or/en/1100068548
- South African Society of Psychiatrists https://www.sasop.co.za/
- World Psychiatric Association https://www.wpanet.org/

## Appendix 3: AGREE II quality appraisal

### Scope and purpose

The percentage scores in the ‘scope and purpose’ domain ranged between 72 and 94%. The CNS guideline scored the highest, with a score of 94%, whereas the NIMHANS guideline scored the lowest with a score of 72%. The main reasons for the variety among guidelines with respect to domain 1 were the differences in clarity with which the aims and questions of the reports were stated.

### Stakeholder involvement

The score range for the ‘stakeholder involvement’ domain was 42–81%. The highest score of 81% was for the BAP guideline, whereas the lowest score of 42% was obtained for the NIMHANS guideline. This was because the guideline did not provide details on the roles of the writers involved in the guideline writing process.

### Rigour of development

Overall, the score range in this domain was 36–91%. The CNS 2020 guideline scored the highest with a score of 91%. Following this was the NICE guideline scoring 88%. The APA, BNS, Canadian, WFSBP, HSSP and BAP guidelines scored 73%, 67%,64%, 60%, 57% and 49%, respectively. The IPS guideline scored the lowest with a score of 36%. Neither BAP nor IPS stated their evidence selection criteria. The BAP guideline's DBS recommendation was based on expert opinion rather than higher-quality evidence. IPS did not state their search strategy or how they formulated their recommendations.

### Clarity of presentation

Overall, the range in this domain was 53–91%. The APA guidelines scored the highest with a score of 92%. Following this was the IPS guideline scoring 89%. The WFSBP, BAP, Canadian, HSSP, CNS and NICE guidelines scored 81%, 78%, 75%, 61%, 61% and 58%, respectively. The BNS guideline scored the lowest with a score of 53%.

### Applicability

Overall, the range in this domain was 19–52%. The IPS guideline scored the highest with a score of 52%. Following this was the APA guideline scoring 44%. The BAP, CNS, HSSP, NICE, WFSBP and Canadian guidelines scored 38%, 33%, 33%, 33%, 31% and 31%, respectively. The BNS guideline was the lowest-scoring guideline with a score of 19%. The majority of the guidelines did not explain facilitators and/or barriers to implementation. None of the guidelines explained the monitoring criteria to measure DBS effectiveness. Only the NICE guidelines involved a health economist for the cost analysis of recommendations.

### Editorial independence

Overall, the range in this domain was 0–100%. The BAP, APA, HSSP, Canadian and CNS guidelines scored the highest with scores of 100% each. Following these were the NICE, BNS and WFSBP guidelines scoring 75% each. The IPS guideline scored the lowest with a score of 0%. IPS did not state their funding or declare competing interests.

## Appendix 4: AGREE II percentage scores per domain for all guidelines

**Table:**

Guideline	Domain 1 (%)	Domain 2 (%)	Domain 3 (%)	Domain 4 (%)	Domain 5 (%)	Domain 6 (%)
The Psychopharmacology Algorithm Project at the Harvard South Shore Program: an algorithm for adults with obsessive–compulsive disorder	89	56	57	83	33	100
Clinical practice guidelines for obsessive–compulsive disorder	72	42	36	89	52	0
Deep brain stimulation for obsessive–compulsive disorder	94	58	91	61	33	100
Deep brain stimulation – depression and obsessive–compulsive disorder.	86	58	67	53	19	75
Canadian Clinical Practice Guidelines for the management of anxiety, post-traumatic stress and obsessive–compulsive disorders	92	67	64	75	31	100
Practice guideline for the treatment of patients with obsessive–compulsive disorder	89	72	73	92	44	100
Deep brain stimulation for chronic, severe, treatment-resistant obsessive–compulsive disorder in adults	83	72	88	58	31	75
Evidence-based pharmacological treatment of anxiety disorders, post-traumatic stress disorder and obsessive–compulsive disorder: a revision of the 2005 guidelines from the British Association for Psychopharmacology	92	81	49	78	38	100
World Federation of Societies of Biological Psychiatry guidelines for treatment of anxiety, obsessive–compulsive and post-traumatic stress disorders – version 3. Part II: OCD and PTSD	83	50	60	81	31	75
